# Alpha 1 Antitrypsin Deficiency, Two Cases of Heterozygous S and Clayton Null Alleles

**DOI:** 10.51894/001c.6382

**Published:** 2017-12-19

**Authors:** Eric M. Rosenbaum, Elisabeth Chapaton-Rivard, Colleen Overdorf

**Affiliations:** 1 Genesys Regional Medical Center, Internal Medicine PGY-3 Resident, Grand Blanc MI; 2 Genesys Regional Medical Center, Pulmonology and Critical Care Physician Grand Blanc, MI; 3 Former Pulmonology and Critical Care Fellow at Genesys Regional Medical Center; currently practicing at Providence St Peter’s Hospital in Olympia, Washington

**Keywords:** emphysema, null allele, clayton, alpha-one antitrypsin

## Abstract

Alpha-1 antitrypsin deficiency (AATD) is a disorder that can lead to early onset lung and liver disease and is considered to be underdiagnosed. The purpose of this paper is to demonstrate the importance of early detection using genotyping of AATD by presenting two very rare cases of this disorder and to remind clinicians to maintain a high level of suspicion for this disorder. Two unrelated patients presented to different pulmonology offices in Grand Blanc, MI and were screened for AATD for different reasons. Testing for both patients included alpha-1 antitrypsin enzyme levels, phenotyping, and genotyping. Both individuals were heterozygotes for S allele and Q0Clayton allele. The Q0Clayton allele is a very rare Null allele that is defined this way because these individuals do not produce any alpha-1 antitrypsin. These cases highlight the need for early testing of patients with risk factors for AATD. Also demonstrated is the need to include genotype testing to accurately identify the risk of developing emphysema and cirrhosis. Lower morbidity and mortality may result if AATD is detected early.

## INTRODUCTION

Alpha-1 Antitrypsin Deficiency (AATD) is an inherited disorder that has been associated with early onset emphysema and liver cirrhosis.^1^ However, some individuals with AATD may not have any clinical manifestations of disease or signs and symptoms may present later in life. This disorder is considered to be largely underdiagnosed^1^ and a targeted screening approach is recommended for its detection in adults.^2^ Chronic obstructive pulmonary disease (COPD) with panacinar emphysema is the most frequent cause of morbidity and mortality in AATD.^1^

While smoking tobacco is a major risk factor for developing COPD in the general population, individuals who smoke and also have AATD have been shown to follow a more rapid disease progression.^3^ COPD typically develops much earlier in these patients, often in the third decade of life, earlier than both nonsmoking individuals with AATD and smokers without.^1^

Liver disease is the second most frequent clinical manifestation of AATD.^1^ It may present as cholestasis in infancy or chronic liver disease in childhood. Deficient adults over the age of 50 are at risk for cirrhosis and hepatocellular carcinoma.^2^ Additional extrapulmonary manifestations of AATD include necrotizing panniculitis, systemic antineutrophil cytosplasmic antibodies (ANCA) positive vasculitis, psoriasis, urticaria, pancreatitis, and angioedema.^1^

The clinical manifestations of this disorder come from mutations in the SERPINA1 gene on chromosome 14 that alter the production of the protein alpha-1 antitrypsin (AAT).^4^ It is one of many serine protease inhibitors and helps to protect lung tissue from neutrophil elastase. Neutrophil elastase is a serine protease, an enzyme that causes breakdown of protein including that of alveolar tissue during inflammatory responses. AAT is primarily synthesized in hepatocytes but also in white blood cells and the cells that line the airway and intestine. This protein is secreted into the serum and functions in tissues with significant neutrophil burden. During an acute phase response, serum levels rapidly increase several-fold.^4^

To date over 100 genetic variants of AAT have been identified. The normal genetic variant or allele is designated M and the two most common deficient variants are the S and Z variants. The Z mutation leads to abnormal folding of AAT as it is produced, thereby altering the way it interacts with other enzymes and molecules. This leads to polymers that form inclusion bodies within hepatocytes unable to be secreted into the serum. Therefore, secretion of the protein into the serum is prevented and it instead accumulates within the liver. This results in not only liver disease but also a significant deficiency in serum AAT,^4^ hence the name of the disease. Lung damage results from the imbalance of neutrophil elastase and AAT. Pulmonary irritants including tobacco smoke and environmental pollutants further raise levels of neutrophil elastase and lead to worsening of the AAT-protease balance.^2^

The risk for emphysema increases as AAT levels decrease below 60 mg/dL (reference range 90-200 mg/dL). Severe cases are often homozygotes with the Z mutation and associated with severe reduction of serum AAT levels ≤ 80%.^5^ The S variant is associated with levels ≤ 60% although it is not associated with significant disease. Furthermore, the S variant leads to a smaller accumulation of polymers in hepatocytes than the Z variant with no clinical consequences.^4^

There is a rare group of AAT alleles that are not detected either during transcription or translation that are referred to as Null alleles. These type of alleles are not associated with liver disease because there is generally no accumulation of polymers within the hepatocytes. A homozygous null allele will lead to development of emphysema even earlier than a ZZ homozygote. It has been estimated that the frequency of all null alleles is about 1/100^th^ that of the Z allele in the North American white population.^4^

### Case Descriptions

Patient One was a Caucasian female in her later 20s who was referred to the pulmonology service for evaluation for AATD due to her strong family history. Her past medical history was significant for uterine cancer status post hysterectomy, uncontrolled type 2 diabetes, liver dysfunction, and obesity. She had never smoked and denied alcohol or illicit drug use. Her father had developed severe emphysema and died at age 49 and her paternal aunt had deficiency but was not currently being treated.

At the time of referral, she had no respiratory complaints and her physical exam was unremarkable. Laboratory data included a slight elevation in ALT and elevated Gamma-Glutamyl Transferase. Her HgbA1C was 11.1%. A pulmonary function test (PFT) showed normal lung function. Immunoassay revealed AAT level of 35.4 mg/dL in the setting of an elevated C-reactive protein (CRP) level of 33 indicating an active inflammatory response. Isoelectric focusing showed a phenotype of SS and genetic sequencing demonstrated that this patient has the S/Q0_Clayton_ genotype.

The patient was counseled concerning the importance of avoiding pulmonary irritants and testing of other family members was encouraged. Since the patient had no concerning pulmonary test results, exam findings, or symptoms, the authors did not initiate treatment for AATD despite her low levels of AAT.

Patient Two was a Caucasian male in his early 50s who was referred to the pulmonology service to evaluate persistent dyspnea of two months duration that became worse with exertion. A cardiac workup was unremarkable. Past medical history included allergic rhinitis, diabetes mellitus, and hypertension. He quit smoking 10 years ago after smoking three packs per day for 15 years. Family history was positive for coronary artery disease. His physical exam was unremarkable. A PFT revealed restrictive changes and no bronchodilatory reversibility although inadequate respiratory effort was noted. His AAT level was 35.1 mg/dL and his CRP was 2.11 mg/dL. Phenotype revealed S band and genotyping revealed S/Q0_Clayton_. This patient was offered enzyme replacement therapy.

## DISCUSSION

The authors completed a literature review and found that the Q0_Clayton_ mutation is very rare. It has only been reported in a U.S. Caucasian family, a Korean family, and a Japanese family.^6,7^ A total of 11 cases of S/Null genotype were found. Of S/Null genotypes two were heterozygotes of Q0_Clayton_ and S_iiyamma_ which is a specific S variant with more severe disease than other S variants. No other cases were described with S and the Clayton null allele.

The DNA sequence for the Q0_Clayton_ allele is a mutation of the normal M1 AAT allele. There is a cytosine insertion that leads to a frameshift mutation causing a premature stop codon at amino acid 374. This cytosine insertion occurs at a location in the DNA sequence that is described as a mutational hot spot due to the many mutations that occur at this location. The resultant protein is smaller and is retained in the rough endoplasmic reticulum where it undergoes degradation prior to translation into AAT.^8^ Because the mRNA is degraded there is no protein production and therefore no retained protein to cause hepatocellular damage.

These cases demonstrate the importance of genetic sequencing of the SERPINA1 gene when testing for AATD. Phenotyping using electrophoresis may lead to an assumption that these patients were homozygous S which has much lower frequency of clinical manifestations. Phenotyping does not detect null alleles. Genotyping allows identification of the null alleles and specific mutations using polymerase chain reaction amplification and sequencing.^9^

These cases also emphasize the synergistic role of tobacco smoke and AATD in causing pulmonary disease. Patient One had significantly deficient levels yet has no pulmonary complaints. These levels of AAT provide decreased protection from inflammatory degradation. We wondered if she would already have evidence of pulmonary disease if she were a smoker or exposed to other irritants. Unfortunately, the natural history of AATD suggests that she may still develop emphysema. Patient Two was a former smoker with current pulmonary symptoms. Patients with AATD who smoke have been found to lose FEV1 at a rate of 70-130 mL per year compared to nonsmokers who lose lung function at 40-70 mL per year indicating a more rapid decline in lung function in smokers.^3^

Patient One had an elevated CRP at the time of testing AAT levels, perhaps indicating an acute inflammatory response. It is important to be aware that AAT levels rise during an acute phase response or exposure to high estrogen states^10^ because this will alter testing for AATD. While Patient One has low levels despite elevated CRP, some patients with AATD may have normal levels of AAT if tested during an acute phase response and may need to be tested after inflammation resolution. This patient may have even lower levels of AAT if CRP is indicative of acute phase response and AAT levels tested after its resolution.

## CONCLUSIONS

In order to intervene and slow the progression of this disease, clinicians need to maintain a high index of suspicion to detect AATD at an early age. Patients with COPD, non-responsive asthma, cryptogenic liver disease, and a family history of AATD in first-degree relatives (e.g., parent sibling or child) should all be screened for AATD. Knowledge of the disorder in deficient patients may provide added incentive to avoid pulmonary irritants.

If patients can quit smoking, change occupations if they are exposed to occupational irritants, and aggressively avoid second-hand tobacco smoke, dusts, and fumes they may experience a slower progression of the disease, lower morbidity and lower mortality. Clinicians should offer these patients preventative measures including vaccinations for influenza, pneumococcus, and hepatitis and undergo hepatic screening. Furthermore, AATD patients will be offered AAT augmentation therapy to slow disease progression. Cigarette smoking is highly addictive and few patients quit successfully. However, patients identified with AATD who do not yet smoke may be less likely to start smoking. In one study, individuals identified with AATD at birth demonstrated lower rates of smoking at adolescence compared to control subjects.^2^

As therapy for AATD advances, early detection will be more important. Full genotype analysis will help prevent false negative results as it did for our patients. We hope that the devastating consequences of this disease may be prevented or at least delayed in these two patients since they were identified early and provided proper counseling.

### Conflict of Interest

The authors declare no conflict of interest.
